# BARRIERS AND OPPORTUNITIES TO HEALTHY AGING IN ANCHORAGE, ALASKA, USING CONCEPT MAPPING

**DOI:** 10.1093/geroni/igz038.1578

**Published:** 2019-11-08

**Authors:** Britteny M Howell, Daniel McLinden

**Affiliations:** 1 University of Alaska Anchorage, Anchorage, Alaska, United States; 2 Idea Networks, LLC, Cincinnati, Ohio, United States

## Abstract

Alaska currently has the fastest growing proportion of older adults than any state in the country, and seniors are choosing to age-in-place in Anchorage in record numbers. Research shows that including older adults with community-based professionals (aging advocates, researchers, service providers) in focus group activities can provide a rich and holistic model of aging that demonstrates a robust foundation for supporting aging and addressing health disparities. This paper presents the results of a project conducted with older adults (50+ years), advocates, and other stakeholders in Anchorage using Concept Mapping (CM) methodology, a technique not often used in the gerontology literature. CM is a mixed-method, participatory approach that uses brainstorming and unstructured card-sorting combined with multivariate statistics (multi-dimensional scaling, hierarchical cluster analysis) to create a data-driven visual representation of thoughts or ideas of a community. CM is well suited to integrating perspectives from multiple points of view. Participants were prompted to address the research question: how do we think about aging in Anchorage & what are the barriers and facilitators to aging well? Results indicate services for seniors should include culturally responsive health programming, low-cost opportunities for social engagement, inclusion of older adults with intellectual/developmental disabilities, transportation considerations, navigators to locate services in Anchorage, and more. CM allowed the researchers to identify how residents view healthy aging in this urban subarctic location and brainstorm practical solutions with stakeholders and local policy-makers. This presentation will also share lessons-learned regarding the use of this participatory approach with older adults.

